# Optimised amino acid specific weighting factors for unbound protein docking

**DOI:** 10.1186/1471-2105-7-344

**Published:** 2006-07-14

**Authors:** Philipp Heuser, Dietmar Schomburg

**Affiliations:** 1 Cologne University Bioinformatics Center (CUBIC), University of Cologne, Zuelpicher Str. 47, 50674 Koeln, Germany

## Abstract

**Background:**

One of the most challenging aspects of protein-protein docking is the inclusion of flexibility into the docking procedure. We developed a postfilter where the grid-representation of proteins for docking is extended by an optimised weighting factor for each amino acid.

**Results:**

For up to 86% of the evaluated complexes a near-native structure was within the top 5% of the ranked prediction output. The weighting factors obtained by the optimisation procedure correlate to a certain extent with the flexibility of the amino acids, their hydrophobicity and with their propensity to be in the interface.

**Conclusion:**

Use of the optimised amino acid specific parameters yields a strong increase of near-native structures on the first ranks of the prediction.

## Background

Protein-protein interactions and complex formation play a central role in a broad range of biological processes, including hormone-receptor binding, protease inhibition, antibody-antigen interaction and signal transduction [1]. As structural genomics projects proceed, we are confronted with an increasing number of structurally known proteins that are functionally uncharacterised. To identify how two proteins are interacting will be particularly important for elucidating functions and designing inhibitors[2]. Although predicting around 50 percent false positive interactions [3], high throughput interaction discovery methods, such as the yeast two hybrid system, suggest thousands of protein-protein interactions and therefore also imply that a large fraction of all proteins interact with other proteins [4].

Since many biological interactions occur in transient complexes whose structures often cannot be determined experimentally, it is important to develop computational docking methods which can predict the structure of complexes with a proper accuracy [5].

Docking algorithms are developed to predict in which orientation two proteins are likely to bind under natural conditions. They can be split in a sampling step followed by a scoring step. A collection of putative structural complexes is generated by scanning the full conformational space in the first step. Afterwards the putative complexes are ranked according to scoring functions based on geometrical and chemical complementarity.

**Figure 1:**
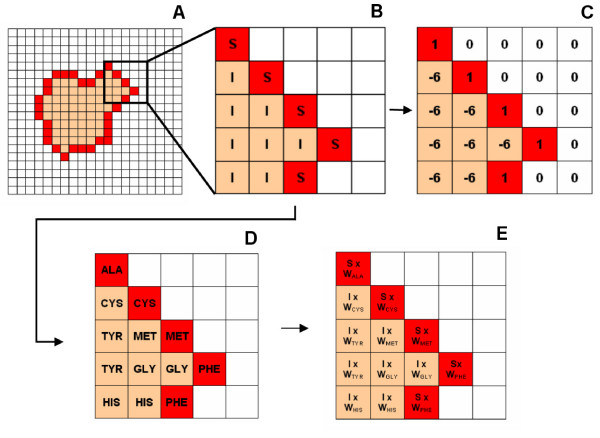
The proteins are mapped on a grid (**A**); In the common representation there is a differentiation between cells representing the surface (S) and the interior (I) (**B**), which results in the distribution of values as shown in **C**. Since each cell also represents the underlying amino acid (**D**), in the new representation (**E**) as it is used here each value is composed of an numerical value for surface and interior (S/I) and a weighting factor for each amino acid (W_AA_).

For the scanning of the conformational space for geometrical complementarity different methods are used (for a general introduction and an overview over the different docking methods see Halperin 2002 [6]). One of the most widely spread docking methods is based on Fast Fourier Transformations (FFT). The usage of FFT was introduced into docking by Katchalsky-Katzir in 1992 [7]. One important aspect of the docking procedure is the representation of the proteins. Most FFT based methods use a grid representation for the proteins [7-11]. Therefore each protein is mapped on a 3D grid, and the cells of the grid get different values assigned, representing the surface or the interior of the proteins (Figure 1A–C). Further grids or complex numbers can be used to represent specific properties which are thought to play a crucial role in protein interactions like hydrophobicity or electrostatics [8,10-12].

During the docking procedure the two grids representing the proteins are moved with respect to each other in a specified number of rotations and the geometric correlation for all translations is calculated in Fourier space within one step. The geometric complementarity of the proteins is evaluated by summing up the products of the values of the overlapping cells. In most approaches the surface cells of the proteins are assigned a value of one. Therefore the more surface cells are in contact with surface cells from the other protein the higher is the geometric score. The interior of the larger protein is assigned a negative value (in our docking program: -6). This results in a 'punishing' negative value as soon as overlaps with interior cells of the first protein are observed. The interior cells of the second protein are assigned a value of one leading to an asymmetrical treatment of both proteins, which 'softens' the surfaces slightly [13].

In the beginning the FFT-based docking methods were developed for *bound docking*. In *bound docking *the complex structure is split in its subunits and the docking algorithm predicts the complex structure from these subunits. For most cases bound docking gives good results, i.e. for most cases a near-native complex structure is on the first rank of the prediction output. However, if the complex structure is to be predicted from the 3D-structures determined in an *unbound *state, most docking procedures do find a near-native solution but not within the first ranks. This can be explained by the conformational differences between the complex and the unbound structures. Therefore the development of *unbound docking *methods, which are able to predict the near-native complex even if conformational changes take place, is the most challenging current task in the field of protein-protein docking.

Each protein-protein interaction depends on the amino acids involved in the interaction. Several attempts to evaluate the importance of the 20 amino acids for protein-protein docking were published [14-18]. Different properties of the amino acids like hydrophobicity, interface propensity, electrostatic properties, flexibility and others were tested for their relevance in docking. Two different approaches were done. On the one hand it was attempted to use these properties to detect the interface region of proteins before the docking procedure [16,19-26] and on the other hand the differences of the amino acids were used to identify the near-native structure of a complex from all those structures showing a high geometrical correlation [27].

However, some of the properties lead to controversial ratings of the amino acids. For example for methionine there is a high propensity to be in the interface of a complex [18], which would lead to an assignment of an important role for that amino acid, but at the same time methionine has a large side chain which might cause clashes in rigid body docking even for the near-native complex which should not be 'punished'.

The flexibility of the amino acid side chains is the main reason for the unsatisfactory results of unbound docking. In the past it was tried to truncate or collapse very flexible side chains like arginine, lysine, asparagine, glutamine and methionine by assigning low numerical values to the cells representing their side chains [10,28,29]. Other approaches to treat the flexibility of side-chains include docking with different copies of the unbound subunits [30], or the usage of rotamer libraries in the refinement step.

Since it is nearly impossible to decide which property and which scale is the best one in each single case, we optimised amino acid specific weighting factors for rigid body unbound-unbound protein-protein docking. Therefore the grid representation of the proteins was extended. The new representation takes the amino acids into account which are represented by the cells. The values assigned to each cell, are composed of a value for surface or interior of the protein and a weighting factor in dependence of the amino acid (Figure 1D–E). These weighting factors were specifically optimised for three different classes of complexes following the classification of the dataset[31]: enzyme-inhibitor/substrate, antibody-antigen and others.

There are two different possible methods to make use of this kind of representation of the proteins. On the one hand these values can be assigned to the cells before the calculation of the geometric correlation. Thereby an improvement of prediction accuracy might be achieved without an extension of the required computation time, but the chosen parameters must be capable of differentiating between clashing complex structures and such which have primarily surface-surface contacts. On the other hand this protein representation can be used to rerank the structures suggested by the calculation of the geometric correlation. This publication is focussed on the second approach.

The aim of our current work is to find new and independent criteria for the reranking of proposed complex structures, which describe other properties of near-native complex structures as our previously published [27] postfilter. An integration of the different approaches described in the literature requires a larger programming exercise but is expecting to give further improvement.

## Results

### Obtained parameter

The weighting factor for each amino acid and the estimated value for all cells of the interior of the receptor obtained by the optimisation are shown for the three different complex classes in Table 1.

There are two groups of amino acids where the obtained parameters are comparable between all three complex classes. On the one hand there is a group consisting of aromatic and hydrophobic amino acids (TRP, TYR, PHE, ILE, VAL) which got high weighting factors for all three complex classes. On the other hand very low parameter were obtained for the amino acids with long and flexible side chains (ARG, GLN, GLU, LYS) and to such amino acids having a very low propensity to be in the interface (ASP, SER, PRO) [18].

For the other amino acids the optimised values differ for the three complex classes. While ALA, ASN and HIS play a major role in finding near-native structures for antibody-antigen complexes, especially MET got a high value for enzyme-inhibitor complexes. For LEU and THR, the optimisation yielded medium values for enzyme-inhibitor complexes and for other complexes but a weighting factor of about null was obtained for the antibody-antigen complexes.

The optimised parameters are specific for the complex class they have been optimised for. The percentage of near-native structures within the top 10% of the reranked output is about 10–15% higher when the parameters obtained from the same complex class are applied as compared to the application of the other parameters.

The values optimised for the interior of the receptor (I1) seriously differ for the three classes. For enzyme inhibitor complexes the value is 8.34, for antibody-antigen complexes -3.45 and for the other complexes 0.15.

### Validation

**Figure 2:**
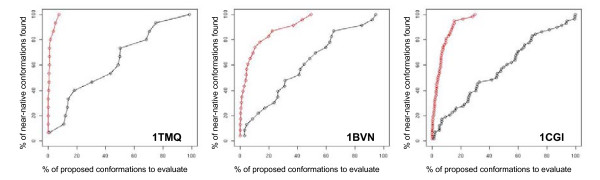
**Enrichment curves for [PDB:1BVN], [PDB:1CGI] and [PDB:1TMQ]**. It is shown how many percent of the dataset must be evaluated until which percent value of all near-native (RMSiCα<5Å) are found. (X-axis: Percentage of dataset, Y-axis: Percentage of near-native solutions found). The black line shows the results for the ranking due to the 'classical' geometric correlation and the red line is obtained by the recalculation using the optimised values. (100% equal for 1BVN 23 near-native structures, for 1CGI 58 and for 1TMQ 15).

For each complex the geometric correlation score was recalculated using the optimised parameter. For most of the complexes a massive enrichment of low RMS-solutions on the lower ranks was achieved. In figure 2 this enrichment is shown for [PDB:1CGI], [PDB:1BVN] and for [PDB:1TMQ]. It is shown how many percent of the dataset must be evaluated until which percent value of all near-native (RMSiCα<5Å) solutions are found. The red line shows the result obtained by the recalculation using the optimised parameter and the black line shows the results before the optimisation.

**Figure 3:**
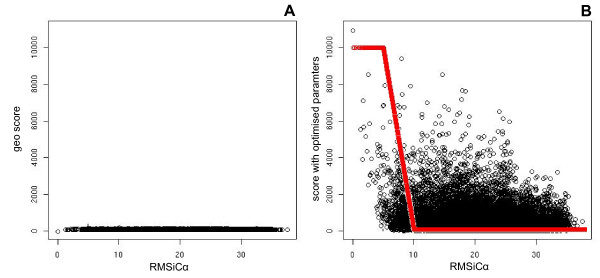
Comparison of the distribution of calculated geometric correlations for a protease/inhibitor example (2SIC) (**A**) without and (**B**) with optimised parameter. The red line in B is the objective function used for the optimisation procedure. Each black circle represents one potential complex structure.

In figure 3 the distribution of the calculated scores is shown. In 3A the calculated geometric correlation compared to the RMS as it is calculated without the optimised amino acid dependent weighting factors is shown, whilst part B shows the same distribution after the optimisation.

**Figure 4:**
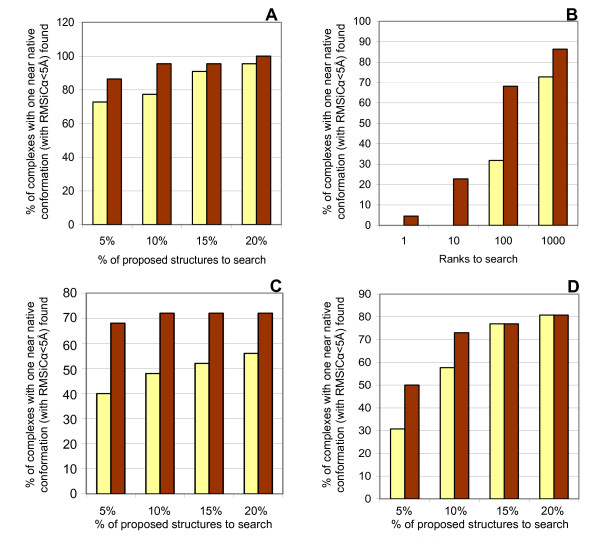
**Improvement of prediction quality (ZDOCK 2 benchmark)**. The light yellow bars show the percentage of complexes for which a near-native solution (RMSiCα<5Å) is found without the optimised weighting factors. The brown bars show the results after the application of the weighting factors. (A/B) for enzyme-inhibitor (C) for antibody-antigen and (D) for other complexes; (100%: 22 enzyme-inhibitor complexes, 25 antibody-antigen complexes and 22 other complexes); an enrichment of complexes with near-native structures on the lower ranks for all complex classes can be seen.

The overall improvement of prediction quality is shown in figure 4. It is shown for how many percent of all tested complexes from the ZDOCK2.0 benchmark a good solution (RMSiCα<5Å) was found within the top ranks.

With non-optimised parameters only 32% of the evaluated enzyme-inhibitor complexes had a solution with an RMS below 5Å within the first 100 ranks. After the optimisation 68% of the complexes do have a good solution within the top 100. For the antibody-antigen complexes the percentage of complexes for which one near- native structure can be found within the top 5% of the ranked predictions increased from 40% to 68% and for the other complexes from 31% to 50%. For the enzyme-inhibitor complexes the percentage of all near-native complexes within the top 5% increased from 13% to 34%, for the antibody-antigen complexes from 8% to 59% and for the other complexes from 6% to 26%.

**Figure 5:**
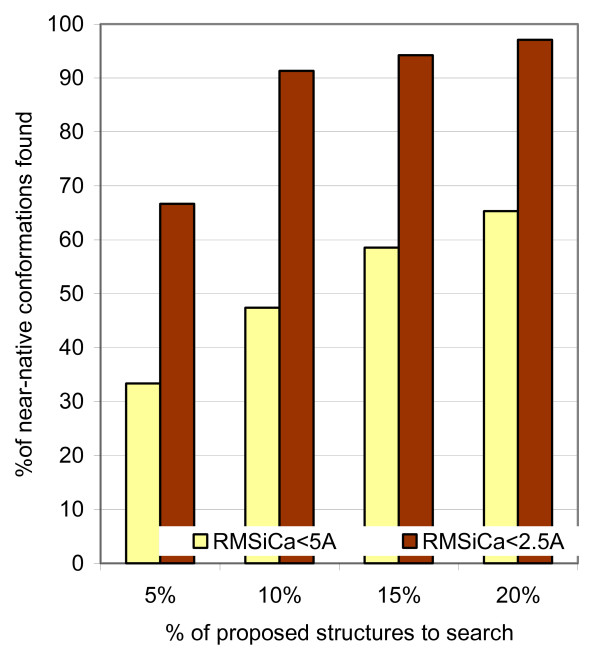
**Enrichment of near-native structures on the lower ranks for enzyme-inhibitor complexes**. The yellow bars show the enrichment for near-native complexes with RMSiCα<5Å and the brown boxes with RMSiCα<2.5Å (100%: 1956 structures with RMSiCα<5Å and 69 structures with RMSiCα<2.5Å).

The application of the optimised weighting factors is especially well suited to recognize those solutions with a very low RMS. Figure 5 shows the enrichment of near-native structures on the lower ranks for structures with RMSiCα<5Å and RMSiCα<2.5Å. For the enzyme-inhibitor complexes more than 90% of all solutions with RMSiCα<2.5Å can be found within the top 10%.

**Figure 6:**
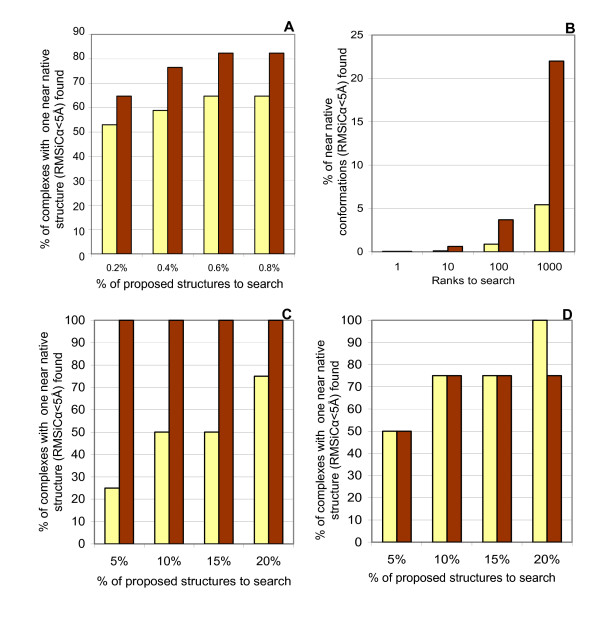
**Improvement of prediction quality (testcases from literature)**. The light yellow bars show the percentage of complexes for which a near-native solution (RMSiCα<5Å) is found without the optimised weighting factors. The brown bars show the results after the application of the weighting factors. (A) for enzyme-inhibitor (100%: 17 complexes) (C) for antibody-antigen (100%: 4 complexes) and (D) for other complexes (100%: 4 complexes), (B) shows the percentage of near-native structures found within the top ranks for enzyme-inhibitor complexes (100%: 1956 structures); Also for the complexes which were not part of the optimisation procedure a clear enrichment of near-native structures on the lower ranks can be seen.

For validation of the optimisation the obtained weighting factors were applied to 17 enzyme-inhibitor, to 4 antibody-antigen and to 4 other complexes from the literature, which are not part of the ZDOCK2 benchmark. Figure 6 shows the improvement achieved for these testcases and table 2 gives a detailed overview over the effect of the reranking on the position of the first and the best near-native structure for each complex.

## Discussion

Although the method is not able to reach the ultimate goal of protein-protein docking, i.e. unambiguously give the near-native geometries the highest scores the increase of near-native structures using the optimised amino acid specific grid values is quite spectacular, especially when the strict measure of 2.5Å RMS from the native geometry is used to be classified as correct. More than 90% of all near-native structures for the enzyme-inhibitor complexes are found within the 10% of the ranked output after rescoring with the optimised grid values. For all three complex-classes the number of near-native complex structures (RMSiCα<5Å) within the top 100 ranks increased by a factor of 3–5.

In comparison to our previously published results [27], this filter performs better than the conservation based filter for those cases, where the conservation based filter was not able to detect the near-native structures reliably (antibody-antigen, several of the enzyme-inhibitor complexes). However, the method described here can not reach the results, which we derived from the domain based post-filter. On the other hand the method described here does not depend on the availability of additional information like homologous complexes.

The optimised parameters comply partially with known properties of amino acids in protein complex interfaces. Amino acids where the optimisation produced very low weighting factors are likely to produce clashes in unbound docking especially for the near-native structures. They would be misleading for the docking of unbound proteins. The lowest values (~0) are assigned to the flexible polar amino acids as ARG, ASP, GLN, GLU or LYS, which also have a very low interface propensity [18]. The high values of the aromatic residues are explainable by their ability to form π-stacks with their ring-systems and by their high propensity to be in interface regions, together with the rigidity of the aromatic ring system.

The weighting factors for the enzyme-inhibitor complexes correspond to the known interface propensities [18], to the number of freely rotatable bonds in the side chain and to the hydrophobicity of the amino acids. The very high value for methionine can be explained by the high propensity to be in the interface and the general rareness of MET on protein surfaces, so that MET gives a strong hint towards the near-native interface region.

The antibody-antigen factors still comply with the above mentioned properties, but to a smaller extend as the factors for the enzyme-inhibitor complexes. Higher values obtained for ASN and TYR correspond to the described higher importance of hydrogen bonds in antibody-antigen complexes.

The set of "other" complexes is rather heterogeneous, so that it is hard to interpretate the optimised values. For these complexes the highest values were also obtained for the apolar, hydrophobic and rigid residues, while for those with long flexible side chains the optimisation ended up with values near null.

Since the number of freely rotatable bonds is correlated with the obtained weighting factors for enzyme-inhibitor complexes, the method presented here is a computational rather cheap way to take the flexibility of amino acid side-chains on the surface of proteins at least partially into account, which is one of the major problems of unbound protein docking methods. The method does not attempt to reproduce the correct conformation of the side chains in the bound state, but it partially ignores the misleading aspect of 'wrong' side chain conformations, which are likely to produce steric clashes even for the near-native conformations in rigid body docking. The correct conformation must be obtained by other methods like energy minimisation procedures.

The method works equally well for complexes classified as "easy" and "medium difficult" [30,31] and also for some bound-cases we evaluated the method is able to enrich the number of near-native structures on the top ranks. This underlines the ability of the filter to identify near-native solutions independently from the flexibility by ignoring clashes of near-native structures.

On the first view the positive value for interior cells of the receptor from the enzyme-inhibitor complexes is surprising since in the common Fourier docking procedure these cells get a negative value assigned. However, it has to be kept in mind that the values were optimised for usage as a postfilter, not for the docking process as such. The structures where the geometrical correlation was recalculated are only those structures obtained by a standard Fourier docking process and therefore have only minor clashes with the interior. All potential complex structures where both proteins are overlapping were excluded in the first calculation of the geometric correlation where -6 was used as a value for the interior cells.

The number of antibody-antigen and of other complexes (4 each) used for validation is quite low due to the lack of available literature values. But the enrichments achieved for the complexes which were also used for the optimisation, gives reason to optimism that the obtained weighting factors will work for the prediction of new antibody-antigen and other complexes as they do for the enzyme-inhibitor complexes.

One of the major advantages of the described method compared to methods that treat side-chain flexibility explicitly consists of the fact that it is computationally much faster.

## Conclusion

The method presented here should work with any rigid body docking system which is based on a grid representation of the proteins. There are, of course, other criteria – like electrostatic interactions, size or hydrophobicity of the interface – that can be used for ranking docking candidates but the work presented here is focused on a rather simple optimisation of the geometric ranking which integrates the different roles of the different amino acids in the interface. The weighting obtained by the optimisation process is partially correlated with different structural properties of the amino acids and takes therefore several aspects into account.

As a next step in this work we will try to find atom-specific weighting factors and combine the method described here with our previously published postfilters and with a comprehensive scoring function (publication in preparation).

## Methods

### The docking tool

In a first step the geometric correlation for 83 unbound protein-protein complexes from the Z-Dock 2 benchmark set[31] was calculated using our docking tool *ckordo *[8,12]. *Ckordo *is a FFT based docking program including further docking arguments such as hydrophobicity and electrostatics. For this work we used only the geometric correlation calculated in Fourier space. The geometric correlation was calculated with a rotation increment of 15° and a maximum cell size of 1.5Å. For each rotation the five structures with the highest geometrical correlation were considered. Since the number of near-native structures is very low compared to the number of incorrect ones additional solutions with low RMS-values were produced by running *ckordo *for 1000 randomly chosen angles in the range from -10° to +10° around the correct rotation. From this run all proposed complex structures with RMSiCα<5Å were selected. This resulted in up to 4700 additional near-native solutions for each complex, which were added to the 22,000 structures calculated with rotational steps of 15°. If the ratio between the number of near-native solutions towards wrong ones is too low, the optimisation procedure would not be able to find the optimal parameters.

For the optimisation process *ckordo *was modified so that for each proposed structure the number of contacts of each amino acid with respect to being surface or interior was calculated. This results in one 20 × 20 matrix for each structure for each possible contact type (surface_protein1 × protein2, interior_protein1 × protein2). Furthermore for each proposed structure the root mean square deviation of the Cα atoms in the interface (RMSiCα) was calculated, in comparison to the unbound proteins fitted on the complex. For the RMSD calculation the Cαs were defined to be part of the interface if at least one atom of the other protein was within a distance of 10Å. The fitting of the unbound proteins on the complex was done with CE [32].

### Optimisation

The contact-matrices and the RMS-values for the complexes from the Z-DOCK 2 Benchmark[31] were used for the optimisation procedure. For each complex class the optimisation was performed independently. It was evaluated if the optimised parameters yield better results if only those structures proposed by the docking tool or those structures and additionally generated near-native complex structures lead to a better result (where the latter was the case). The optimisation was done using the nonlinear minimisation method (nlm()) from the R-package for statistical computing [33]. The formula which is used for the calculation of the geometric correlation until now equals F1. For the optimisation procedure the formula was extended by the weighting factors (F2).

**Figure 7:**
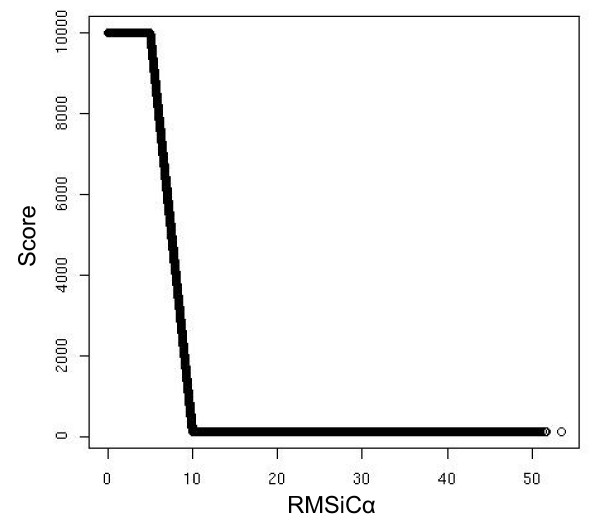
Model for the quadratic error minimisation.

The optimisation itself is a minimisation of the quadratic error between an objective function and the scores obtained. In a preliminary step several models such as logarithmic and linear scales were evaluated as objective function using a small subset of the examples. Finally the best results were obtained for the function shown in Figure 7. All near-native structures (RMSiCα: 0–5Å) were assigned a 100 times

F1

*geoscore *= ∑(*P*1*xP*2)

**Formula 1: **Formula for the calculation of the geometric score. The values in the overlapping grid cells of protein 1 (P1) and protein 2 (P2) multiplied and summed up.

F2

*weighted_geo_score *= ∑((*W*_*AA*_*xP*1)*x*(*W*_*AA*_*xP*2))

**Formula 2: **Formula for the calculation of the geometric score after inclusion of the amino acid specific weighting factors. (P1 = cells of protein1, P2 = cells of protein2, WAA = weighting factor for amino acid)

higher numerical value (10,000) as those showing a RMS value higher than 10Å. For those structures between near-native and 'wrong' structures (RMSiCα: 5–10Å) the target values were calculated by a linear function.

It was evaluated if there is an improvement in prediction quality when the values for surface and for interior were included in the optimisation procedure. It turned out that the best results were achived, when only the value for the interior of the larger protein – usually the receptor – is included in the optimisation and the value for the other protein was kept fixed at the value of 1.

The nonlinear minimisation function of the R-package [33] uses a Newton-type algorithm [34,35]. This method allows finding a minimum of a function by numerical computation of the derivatives. As a convergence criteria for the optimisation the default parameter were used.

### Validation

Due to hardware limits it was impossible to use all available structures for the optimisation, so that subsets had to be chosen. To prove that several different subsets lead to similar results a 5-fold cross validation procedure was performed. Therefore the different complexes from each class were grouped randomly in 5 groups. The optimisation was run 5 times each time leaving out one of the groups and optimising with the remaining four. The final results were calculated using the average value of the five optimisations.

Furthermore the effect of the obtained parameters was evaluated on 17 enzyme-inhibitor, 4 antibody-antigen and 4 other complexes from literature[36], which were not part of the training. The docking procedure for these testcases was run with a rotation increment of 12° leading to 43080 potential structures for each complex. The evaluation was done with respect to the number of complexes which do have a near-native solution within the top ranks and to the number of near-native structures on the first ranks.

## Authors' contributions

The described results are obtained by a combined effort of the two authors where PH did the actual computer work including programming and integration of the method whereas the method was developed and results were discussed in frequent discussions between the two authors. The original idea was from DS.

## Figures and Tables

**Table 1:** Amino acid specific weighting factors. Average optimised parameter for all amino acids obtained from the 5 cross validation runs for three different classes of protein complexes.

**Amino Acid**	**Antibody/Antigen**	**Enzyme/Inhibitor**	**Others**
**ALA**	2.32	0.68	0.74
**ARG**	0.00*	1.70	1.09
**ASN**	5.23	0.49	0.92
**ASP**	0.00*	0.00*	0.00*
**CYS**	0.14	1.11	0.18
**GLN**	0.00*	0.00*	1.06
**GLU**	0.15	0.00*	0.00*
**GLY**	2.49	1.47	7.24
**HIS**	8.74	3.17	0.00*
**ILE**	6.83	2.30	4.34
**LEU**	0.02	3.24	4.83
**LYS**	0.67	0.59	1.29
**MET**	1.46	9.63	3.90
**PHE**	4.46	3.14	7.89
**PRO**	1.98	0.00*	1.13
**SER**	0.00*	0.00*	0.00*
**THR**	0.25	2.67	3.08
**TRP**	4.99	5.19	2.56
**TYR**	11.64	4.94	8.83
**VAL**	3.10	2.78	3.02
**I1**	-3.45	8.34	0.15

* None of these values is exactly 0.0. The optimised weighting factors for these amino acids are between 0.0001 and 0.0008

**Table 2:** Ranks of near-native structures using the optimised weighting factors

	**First structure with RMS<5Å in the ranking**				**Best structure with RMS<5Å in the ranking**			
	**optimised**		**unoptimised**		**optimised**		**unoptimised**	

	**Rank**	**RMS**	**Rank**	**RMS**	**Rank**	**RMS**	**Rank**	**RMS**

**EI**								
**1ACB**	3	2.638	19	0.934	91	0.934	19	0.934
**1AVW**	200	4.864	1024	2.978	2684	1.591	31428	1.591
**1BRC**	15	4.564	10	4.373	3118	1.019	27685	1.019
**1BRS**	5	1.966	35	4.685	5	1.966	22544	1.966
**1BVN**	2332	4.416	116	3.978	4118	1.718	15554	1.718
**1CGI**	31	4.295	57	4.616	922	1.659	27836	1.659
**1CHO**	1	2.779	63	0.809	171	0.809	63	0.809
**1CSE**	4334	4.791	414	3.526	9756	0.614	5831	0.614
**1DFJ**	532	4.145	6441	4.145	5182	3.869	17621	3.869
**1FSS**	149	4.098	228	1.621	467	1.092	966	1.092
**1MAH**	94	2.442	380	4.878	508	1.183	840	1.183
**1PPF**	9	4.351	37	4.593	242	2.457	23526	2.457
**1TGS**	2	1.97	59	3.366	848	0.849	169	0.849
**1UGH**	13	2.672	45	3.031	33	2.023	17059	2.023
**2KAI**	18	3.959	1	4.873	1387	1.316	28310	1.316
**2MTA**	374	4.64	110	4.743	3704	1.111	32445	1.111
**2PCB**	881	4.419	1803	4.419	11627	3.184	26851	3.184
**2PCC**	2548	4.125	1122	2.885	24009	2.885	1122	2.885
**2PTC**	20	4.572	558	4.428	8695	1.439	11164	1.439
**2SNI**	59	3.733	538	4.63	2701	2.814	3803	2.814
**OTH**								
**1AVZ**	19685	3.964	7706	3.964	20034	3.891	11460	3.891
**1BDJ**	2216	4.552	7	2.799	39913	1.986	9733	1.986
**1L0Y**	839	2.271	4103	4.747	19996	1.627	29097	1.627
**1WQ1**	305	2.756	6	4.75	608	1.711	23213	1.711
**AA**								
**1AHW**	580	4.766	143	1.943	620	1.07	4722	1.07
**1DQJ**	1066	3.966	6580	3.966	1066	3.966	6580	3.966
**1VFB**	323	4.894	3706	4.683	2763	1.481	35146	1.481
**1WEJ**	304	4.025	11382	4.025	789	0.917	34658	0.917

## References

[B1] Valdar WS, Thornton JM (2001). Protein-protein interfaces: analysis of amino acid conservation in homodimers. Proteins.

[B2] Caffrey P (2003). Conserved amino acid residues correlating with ketoreductase stereospecificity in modular polyketide synthases. Chembiochem.

[B3] Deane CM, Salwinski L, Xenarios I, Eisenberg D (2002). Protein interactions: two methods for assessment of the reliability of high throughput observations. Mol Cell Proteomics.

[B4] Aloy P, Russell RB (2002). The third dimension for protein interactions and complexes. Trends Biochem Sci.

[B5] Vajda S, Camacho CJ (2004). Protein-protein docking: is the glass half-full or half-empty?. Trends Biotechnol.

[B6] Halperin I, Ma B, Wolfson H, Nussinov R (2002). Principles of docking: An overview of search algorithms and a guide to scoring functions. Proteins.

[B7] Katchalski-Katzir E, Shariv I, Eisenstein M, Friesem AA, Aflalo C, Vakser IA (1992). Molecular surface recognition: determination of geometric fit between proteins and their ligands by correlation techniques. Proc Natl Acad Sci U S A.

[B8] Meyer M, Wilson P, Schomburg D (1996). Hydrogen bonding and molecular surface shape complementarity as a basis for protein docking. J Mol Biol.

[B9] Ackermann F, Herrmann G, Posch S, Sagerer G (1998). Estimation and filtering of potential protein-protein docking positions. Bioinformatics.

[B10] Chen R, Weng Z (2002). Docking unbound proteins using shape complementarity, desolvation, and electrostatics. Proteins.

[B11] Gabb HA, Jackson RM, Sternberg MJ (1997). Modelling protein docking using shape complementarity, electrostatics and biochemical information. J Mol Biol.

[B12] Zimmermann O (2002). Untersuchungen zur Vorhersage der nativen Orientierung von Protein-Komplexen mit Fourier-Korrelationsmethoden. Institute for Biochemistry.

[B13] Eisenstein M (2004). Introducing a 4th dimension to protein-protein docking. Structure (Camb).

[B14] Jones S, Thornton JM (1996). Principles of protein-protein interactions. Proc Natl Acad Sci U S A.

[B15] Jones S, Thornton JM (1997). Analysis of protein-protein interaction sites using surface patches. J Mol Biol.

[B16] Lichtarge O, Bourne HR, Cohen FE (1996). An evolutionary trace method defines binding surfaces common to protein families. J Mol Biol.

[B17] Lo Conte L, Chothia C, Janin J (1999). The atomic structure of protein-protein recognition sites. J Mol Biol.

[B18] Chakrabarti P, Janin J (2002). Dissecting protein-protein recognition sites. Proteins.

[B19] Jones S, Thornton JM (1997). Prediction of protein-protein interaction sites using patch analysis. J Mol Biol.

[B20] Fernandez-Recio J, Totrov M, Skorodumov C, Abagyan R (2005). Optimal docking area: a new method for predicting protein-protein interaction sites. Proteins.

[B21] Pupko T, Bell RE, Mayrose I, Glaser F, Ben-Tal N (2002). Rate4Site: an algorithmic tool for the identification of functional regions in proteins by surface mapping of evolutionary determinants within their homologues. Bioinformatics.

[B22] Landgraf R, Xenarios I, Eisenberg D (2001). Three-dimensional cluster analysis identifies interfaces and functional residue clusters in proteins. J Mol Biol.

[B23] Fariselli P, Pazos F, Valencia A, Casadio R (2002). Prediction of protein--protein interaction sites in heterocomplexes with neural networks. Eur J Biochem.

[B24] Zhou HX, Shan Y (2001). Prediction of protein interaction sites from sequence profile and residue neighbor list. Proteins.

[B25] Koike A, Takagi T (2004). Prediction of protein-protein interaction sites using support vector machines. Protein Eng Des Sel.

[B26] Bordner AJ, Abagyan R (2005). Statistical analysis and prediction of protein-protein interfaces. Proteins.

[B27] Heuser P, Bau D, Benkert P, Schomburg D (2005). Refinement of unbound protein docking studies using biological knowledge. Proteins.

[B28] Palma PN, Krippahl L, Wampler JE, Moura JJ (2000). BiGGER: a new (soft) docking algorithm for predicting protein interactions. Proteins.

[B29] Heifetz A, Eisenstein M (2003). Effect of local shape modifications of molecular surfaces on rigid-body protein-protein docking. Protein Eng.

[B30] Zacharias M (2003). Protein-protein docking with a reduced protein model accounting for side-chain flexibility. Protein Sci.

[B31] Mintseris J, Wiehe K, Pierce B, Anderson R, Chen R, Janin J, Weng Z (2005). Protein-Protein Docking Benchmark 2.0: an update. Proteins.

[B32] Shindyalov IN, Bourne PE (1998). Protein structure alignment by incremental combinatorial extension (CE) of the optimal path. Protein Eng.

[B33] R Development Core Team (2005). R: A language and environment for statistical computing.

[B34] Dennis JE, Schnabel RB (1983). Numerical Methods for Unconstrained Optimization and Nonlinear Equations. Prentice-Hall, Englewood Cliffs, NJ.

[B35] Schnabel RB, Koontz JE, Weiss BE (1985). A modular system of algorithms for unconstrained minimization.. ACM Trans Math Software.

[B36] Heuser P, Martin O UUPPDD - Unbound Unbound Protein Protein Docking Dataset. http://biotool.uni-koeln.de:8080/uuppdd/.

